# Evaluating Safety and Efficacy of Bio‐Immune (Andrographolide) in Managing URTI Symptoms: A Randomized, Double‐Blinded, Placebo‐Controlled, Single‐Center, Comparative Study

**DOI:** 10.1155/tswj/2916630

**Published:** 2026-09-18

**Authors:** Shashi Chandrama Singh, Muskan Choudhary, Khushi Choudhary, Harsh Pal Singh

**Affiliations:** ^1^ Ambe Phytoextracts Pvt. Ltd., Research and Development Centre, Pauri, Uttarakhand, India, ncbir.pl

**Keywords:** 2-hydroxy beta cyclodextrin, *Andrographis paniculata*, andrographolide, Bio-Immune, common cold, cough, health supplement, immunity, nutraceutical, upper respiratory tract infection

## Abstract

**Introduction:**

Bio‐Immune is a standardized extract containing andrographolide, a diterpenoid lactone derived from the aerial parts of *Andrographis paniculata* (commonly known as Kalmegh), and the carrier, 2‐hydroxypropyl beta‐cyclodextrin. Kalmegh has long been used to treat uncomplicated upper respiratory tract infection (URTI) in complementary and alternative medicine. A randomized, double‐blind, placebo‐controlled clinical trial was conducted to evaluate the efficacy of Bio‐Immune (≥ 17% *w*/*w* andrographolide content).

**Methods:**

Participants were randomized to receive either 100 mg Bio‐Immune twice daily or placebo for a 5‐day intervention period (Clinical trial registration number: CTRI/2024/12/077632, Registered 4, December 2024). The primary outcome measure was the Wisconsin Upper Respiratory Symptom Survey‐21 (WURSS‐21) score; secondary outcomes included Visual Analogue Scale (VAS) and Numeric Rating Scale (NRS) scores, nasal mucus weight, C‐reactive protein (CRP), Interleukin‐8 (IL‐8), and Immunoglobulin A (IgA) in nasal wash, along with safety.

**Results:**

Of 67 screened participants, 56 were enrolled, and 55 completed the study. The WURSS‐21, VAS, and NRS scores in the Bio‐Immune group improved, with statistically significant reductions suggesting relief from symptoms associated with uncomplicated URTI and sustained functional improvement owing to Bio‐Immune supplementation. Although the placebo group showed gradual improvement over time, the speed and magnitude of recovery were consistently and statistically superior in the Bio‐Immune cohort, with significant changes evident by 6 h after supplementation. Furthermore, a significant reduction in nasal mucus weight was observed by Day 5, accompanied by minor downward trends in CRP and IL‐8, which lacked adequate statistical support. No adverse events were reported during the intervention period.

**Conclusions:**

Clinical findings support the efficacy of Bio‐Immune at a lower dose in providing URTI symptom relief compared with placebo in a shorter intervention period.

**Trial Registration:**

Clinical Trial Registry of India: CTRI/2024/12/077632; ClinicalTrials.gov identifier: NCT06689995

## 1. Introduction

Uncomplicated upper respiratory tract infections (URTIs) are typically characterized by nasal obstruction or congestion, cough, sore throat, and general physical discomfort. URTIs involve an acute inflammatory response in the upper respiratory mucosa, typically presenting with or without cough, without clinical signs of pneumonia, no identifiable alternative diagnosis, and no prior history of chronic obstructive pulmonary disease, emphysema, or chronic bronchitis [1–3]. Most uncomplicated URTIs are self‐limiting and typically resolve within 1–2 weeks; however, they are often associated with significant physical discomfort and impairment of daily activities. Treatment is primarily focused on alleviating symptoms and accelerating recovery in shorter durations, allowing individuals to maintain their daily activities without disruption.

A variety of herbs have been used for centuries in treating respiratory ailments, owing to their richness in bioactive compounds that contribute to therapeutic effects. *Andrographis paniculata* (Burm. f.) Wall. ex Nees has long been recognized as a natural remedy for uncomplicated URTIs, owing to its anti‐inflammatory, antimicrobial, antiviral, and immunomodulatory properties [4, 5].

*Andrographis paniculata* has been extensively studied for its efficacy in managing the clinical symptoms of URTIs, including nasal obstruction, cough, sore throat, common cold, and other associated symptoms [6]. The health benefits of *A. paniculata* in the treatment of common cold and URTIs have been demonstrated in numerous clinical studies. A placebo‐controlled, double‐blind clinical trial demonstrated the beneficial effects of *A. paniculata* extract in participants with common colds. The participants were divided into two groups, in which Group 1 (*n* = 33) received 1200 mg of *A. paniculata* extract in tablet form, whereas Group 2 (*n* = 28) received a placebo. A significant reduction in clinical symptoms at Day 4 was observed compared with placebo [7]. In this study, the dose of *A. paniculata* extract was quite high. In another randomized, double‐blind, placebo‐controlled clinical trial, the efficacy *of A. paniculata* extract (200 mg/day, containing > 30% *w*/*w* andrographolide) was evaluated in participants with URTIs using the Visual Analogue Scale (VAS), and the extract was found to be effective in reducing URTI symptoms within 5 days [8]. In this study, the limitation was that only the VAS score was used to evaluate the efficacy of the herb extract. Another study was conducted with different doses of *A. paniculata*, assessed by the Wisconsin Upper Respiratory Symptom Survey (WURSS‐21) in comparison with those receiving a placebo for the 7‐day intervention period. *Andrographis paniculata* 200 and 400 mg/day (contains andrographolide > 30% *w*/*w*) in comparison with placebo significantly managed common cold symptoms with no safety concerns on Day 3 [3].

Bio‐Immune is a standardized complex of andrographolide‐2‐hydroxypropyl‐*β*‐cyclodextrin (AND‐2‐HPBCD) that improves the solubility, bioavailability, and half‐life of andrographolide, the active molecule [9]. The process of Bio‐Immune preparation has been patented in India (2025). Andrographolide, a diterpenoid lactone, is the primary active therapeutic moiety found in the aerial parts of *A. Paniculata* [10], whereas 2‐hydroxypropyl‐*β*‐cyclodextrin (2‐HPBCD) serves as the active carrier. 2‐HPBCD, a derivative of cyclodextrin, is widely used to enhance the biopharmaceutical properties of compounds via both oral and inhalation routes of administration [11]. A 1.5‐fold increase in the absorption of andrographolide was observed after complexation with 2‐HPBCD, along with improvements in the bioavailability and half‐life of andrographolide. Bio‐Immune has been reported to possess antiviral activity against Severe Acute Respiratory Syndrome Coronavirus 2 in in vitro preclinical studies [9].

Toxicity analysis (acute and subacute) of Bio‐Immune was conducted in Sprague–Dawley rats for both oral and inhalation routes of administration. In the single‐dose acute oral toxicity study, LD_50_ (lethal dose) was found to be > 2000 mg/kg, and in addition, in a subacute oral toxicity study over a period of 28 days under repeated‐dose oral toxicity study, no observed adverse effect level (NOAEL) was found to be 666 mg/kg. Correspondingly, single‐dose acute inhalation toxicity of Bio‐Immune was carried out at 5 mg/L/4 h/day and subacute inhalation toxicity at 0.5, 1, and 1.66 mg/L/4 h/day over a period of 28 days. The NOAEL was estimated to be 0.5 mg/L/4 h/day [12].

The primary objective of this study was to assess the efficacy of Bio‐Immune at a low dose of 100 mg (containing ≥ 17% *w*/*w* andrographolide—active content), twice a day, in reducing the symptoms of URTIs, as measured by the WURSS‐21, a validated scale, in comparison with a placebo over a 5‐day intervention period. The secondary objectives were to assess certain objective efficacy measures (total VAS and Numeric Rating Scale [NRS] score, nasal mucus weight, C‐reactive protein [CRP], Interleukin‐8 [IL‐8], and Immunoglobulin A [IgA]), as well as safety when compared with placebo.

## 2. Methods

### 2.1. Participants and Study Design

This clinical trial was designed as a pilot, exploratory study to evaluate the preliminary efficacy, safety, and tolerability of the product at a low dose of andrographolide, as well as to gather data to power future definitive trials.

A total of 67 participants were screened, with a 10% dropout rate, resulting in 56 participants (28 participants per group) with uncomplicated URTIs who completed the study. The sample size was chosen based on pragmatic considerations for an exploratory pilot trial, where a minimum sample size of 50 participants (25 in each study arm) was deemed sufficient to assess feasibility, evaluate variance, and determine effect sizes for future larger scale clinical investigations. Eligible participants were aged 30–80 years and presented with symptoms such as cough, nasal discharge, sore throat, or a fever spike within 48 h of enrolment. This specific age baseline was chosen for this pilot evaluation to secure a highly stable and occupationally consistent cohort, minimizing the immune and lifestyle variance frequently observed across younger demographics. Inclusion and exclusion criteria are outlined in Table 1.

**Table 1:** Inclusion and exclusion criteria.

**Inclusion criteria**	1. The age of the participants was ≥ 30 years and < 80 years.
	2. The participant was male or female (nonpregnant and nonlactating) gender.
	3. The participant was suffering from uncomplicated URTI characterized by symptoms such as cough, nasal discharge, and sore throat or had the first fever spike within 48 h of enrolment.
	4. The participant was willing to comply with all study procedures and restrictions, including taking the test treatment as directed, completing the WURSS‐21 questionnaire, and undergoing laboratory assessments.
	5. The participant provided written informed consent prior to participation in the study.
	6. The participant was in a stable medical condition, not requiring immediate intervention or hospitalization.
	7. If the participant was female, she was willing to use a highly effective method of contraception throughout the clinical investigation.
	a. Females of childbearing potential practiced and maintained an established method of birth control (e.g., IUD, hormonal implant device/injection, birth control pills, diaphragm, condoms with spermicide, partner vasectomy, or abstinence).
	b. Nonchildbearing potential females who were surgically sterile, were postmenopausal for at least 1 year or had a tubal ligation, had been using hormonal contraception for at least 6 months, and agreed to continue using the same contraception for the study duration.

**Exclusion criteria**	1. The participant was diagnosed with active respiratory infections or diseases other than uncomplicated URTI that might have required immediate medical attention or intervention and was excluded.
	2. The chest X‐ray of the participant, performed within the past 28 days, revealed significant respiratory disorders or other serious conditions that might have interfered with the study or necessitated medical intervention.
	3. Laboratory tests (blood and urinalysis) performed at the screening visit revealed significant infective or other serious conditions that could have interfered with the study or necessitated medical intervention.
	4. The participant had known immunocompromising conditions such as HIV/AIDS or was undergoing immunosuppressive therapy.
	5. The participant had other significant respiratory diseases (e.g., COPD, asthma, interstitial lung disease, or active tuberculosis).
	6. The participant had uncontrolled or severe cardiovascular, renal, or hepatic conditions.
	7. The participant had participated in any other clinical trial within 30 days prior to the screening visit.
	8. The participant was pregnant/lactating or was planning to become pregnant during the course of the study.
	9. The participant had known hypersensitivity or allergies to any component of the test treatment or similar botanical extracts and was excluded.
	10. The participant was on regular medications known to interfere with the study outcomes (e.g., systemic corticosteroids or antiviral drugs) within 4 weeks before screening and was excluded.
	11. The participant had any condition that, in the investigator′s judgment, would have compromised the participant′s safety or study integrity.

This study was conducted in accordance with the Code of Ethics of the World Medical Association (Declaration of Helsinki) for experiments involving human participants and was approved by the Clinic Research Ethics Committee—ACEAS Independent Ethics Committee registered at CDSCO and OHRP US DHHS, with CDSCO registration number ECR/281/Indt/GJ/2017/RR‐21 and OHRP US DHHS registration number IRB00011046.

This clinic trial is registered at the Clinical Trial Registry of India (CTRI) under the registration number CTRI/2024/12/077632 (Registered on 4, December 2024) and at Clinicaltrials.gov (registry of clinical trials, run by the United States National Library of Medicine [NLM] at the National Institutes of Health [NIH]), with the ClinicalTrials.gov Identifier: NCT06689995. This clinical study was conducted at a site: Novo Bliss Research Pvt. Ltd., Gandhinagar, Gujarat, India. All participants provided written informed consent before enrolment.

The primary endpoint of the study was to evaluate the efficacy of Bio‐Immune in alleviating URTI symptoms, including cough, expectoration, nasal discharge, headache, fever, sore throat, earache, and malaise/fatigue, compared with a placebo over a shorter duration. Symptom severity and functional impairment were assessed using the WURSS‐21 questionnaire, with evaluations conducted on Day 1 (preadministration), 6 h after dose, and follow‐ups on Days 2, 3, and 5. The overall symptom burden was quantified using the area under the curve (AUC) for WURSS‐21 scores.

The secondary endpoint included evaluating the functional effects of Bio‐Immune on symptom relief using the VAS and NRS across all visits, as well as assessing inflammatory and immune biomarkers. Objective assessments included nasal mucus weight recorded using preweighed tissues on Days 1, 2, 3, and 5 to measure congestion relief. Biomarker analyses, including CRP levels in blood, were conducted on Days 1 and 3, along with IL‐8 and IgA levels in nasal wash samples, to assess the intervention′s impact on inflammation and immune response.

The safety endpoint was monitored through continuous adverse event reporting and laboratory investigations. Hematological parameters were assessed, including complete blood count (CBC), and biochemical assessments such as serum creatinine, serum glutamic‐pyruvic transaminase (SGPT), serum glutamic‐oxaloacetic transaminase (SGOT), lipid profile, random blood sugar (RBS), uric acid, and urinalysis. Any deviations from normal ranges were documented and evaluated for clinical significance.

Participants were randomly assigned to two groups: Treatment Arm A (*n* = 28) and Treatment Arm B (*n* = 28), and were instructed to take Bio‐Immune 100 mg twice a day or a placebo. Participants in each group were instructed to take a Bio‐Immune capsule or a placebo twice a day after meals for 5 consecutive days.

The Bio‐Immune capsule contained andrographolide (≥17%*w*/*w*) extracted from *A. paniculata* and an active carrier, 2‐HPBCD, whereas the placebo capsule contained 2‐HPBCD only. The appearance and packaging of the placebo capsule were consistent with those of Bio‐Immune.

The dose was selected based on the available literature, with 200 mg chosen to evaluate the safety and efficacy of Bio‐Immune over a shorter duration. Paracetamol, not exceeding 4 g/day, as and when required, was given as rescue medication.

### 2.2. Outcome Measures

The WURSS‐21 is a validated questionnaire used to assess the effect of uncomplicated URTIs on participants. The WURSS‐21 captures the severity of subjective symptoms of URTIs (e.g., sore throat, cough, and nasal congestion) and assesses quality of life, such as how symptoms interfere with daily activities like sleeping, breathing, concentration, work, and social interaction. In this study, WURSS‐21 was used as a primary outcome measure to evaluate changes in symptom severity and functional impact in participants with uncomplicated URTIs. Assessments were conducted on Day 1 (preadministration), 6 h after dose on Day 1, and follow‐ups on Days 2, 3, and 5.

The WURSS‐21 questionnaire was used to assess symptom severity (Questions 2–11), which evaluated symptoms such as runny nose, nasal congestion, sneezing, sore throat, scratchy throat, cough, hoarseness, head congestion, chest congestion, and fatigue. Each symptom was rated on a 7‐point scale, where “0” indicated “*no symptom*” and “7” indicated “*severe symptoms*.” The functional impairment score (Questions 12–20) assessed the impact of symptoms on daily functioning, including the ability to think clearly, sleep well, breathe easily, walk or climb stairs, exercise, perform daily activities, work inside and outside the home, interact with others, and manage personal life. This was also rated on a 7‐point scale. The AUC was calculated for each score over the 5‐day period from the start of the intervention, representing the cumulative impact of the symptom burden on participants suffering from URTIs. AUC was computed for the symptom score (derived from Questions 2–11), functional impairment score (derived from Questions 12–20), and global score (derived from Questions 2–20). The WURSS‐21 (Copyright 2004 by Bruce Barrett et al., University of Wisconsin–Madison Department of Family Medicine. All rights reserved by the Wisconsin Alumni Research Foundation [WARF]) questionnaire used in this study was licensed from WARF. The following were the secondary outcome measures.

#### 2.2.1. Secondary Outcome Measures

##### 2.2.1.1. Evaluation of URTI Symptoms Using the VAS

The VAS was used to assess the overall severity of symptoms associated with URTI, including cough, expectoration, nasal discharge, headache, fever, sore throat, and earache. The VAS was a 100‐mm linear scale, with endpoints representing the extremes of symptom severity. Participants were asked to mark a point on the line that reflected their perception of overall symptom intensity, with “0” indicating “*no symptoms*” and “100” representing the “*worst imaginable symptoms*.” The distance (in mm) from the “no symptoms” end to the participant′s mark was measured to quantify the symptom intensity. This provided a continuous variable that was statistically analyzed to assess changes in symptom severity over time. VAS scores were categorized as follows: 0–4 mm, no symptoms; 5–44 mm, mild symptoms; 45–74 mm, moderate symptoms; and 75–100 mm, severe symptoms. Assessments were conducted on Day 1 (preadministration), 6 h after administration on Day 1, and follow‐ups on Days 2, 3, and 5.

##### 2.2.1.2. Evaluation of URTI Symptoms Using the NRS

The NRS was used to assess the severity of symptoms associated with uncomplicated URTI, including cough, expectoration, nasal discharge, headache, fever, sore throat, and earache. The NRS is an 11‐point scale, ranging from 0 to 10, where “0” indicates “*no symptom*” and “10” represents the “*worst possible symptom*.” Assessments were conducted on Day 1 (preadministration), 6 h after administration on Day 1, and follow‐ups on Days 2, 3, and 5.

##### 2.2.1.3. Evaluation of Nasal Mucus Weight and Biomarker Parameters

Nasal mucus weight was evaluated as an objective measure to assess the severity of illness associated with URTI. A nasal mucus weighing kit, consisting of preweighed tissues and plastic bags with zip‐lock seals, was provided to participants in advance. Participants were instructed to use the tissues to clear nasal secretions and collect them in the provided bags. Study staff collected the sealed bags, along with any unused tissues, on the day of observation. The total nasal mucus weight was calculated by subtracting the weight of the empty plastic bag and unused tissues from the total collection weight. This evaluation was conducted on Day 1 (preadministration), Day 2, Day 3, and Day 5 to objectively track mucus production throughout the study.

Changes in CRP, IL‐8, and IgA in nasal wash at baseline and Day 3 were also measured.

### 2.3. Randomization and Blinding Procedures

Randomization was employed in this two‐arm, double‐blind study. Participants were randomly assigned in a 1:1 ratio to receive either Bio‐Immune or placebo capsules. To ensure strict allocation concealment, the randomization code was generated by the Biostatistics and Programming Department of NovoBliss Research (CRO). Double‐blind conditions were strictly maintained, with neither the assessor nor the investigator aware of the treatment allocation. The study was conducted using a double‐blind design. Participants were randomly assigned to either Bio‐Immune or a placebo capsule, respectively, according to the randomization code. Double‐blind conditions were strictly maintained, with neither the assessor nor the investigator aware of the treatment allocation. To preserve the integrity of the double‐blind design, the placebo capsules were manufactured to be entirely identical to the active Bio‐Immune capsules in terms of size, shape, weight, color, texture, and odor. Study staff involved in product dispensing and distribution did not engage in any other study‐related activities. Additionally, participants remained blinded to whether they had received Bio‐Immune or placebo capsules.

### 2.4. Allotment of Participant ID

At the time of screening, each participant was assigned a screening number, which appeared on the informed consent document, case report form, and all other study‐related documents. Only participants who passed the screening process were considered for inclusion in the study. Once a participant′s eligibility was confirmed, they were assigned a unique participant number according to the test facility′s guidelines and procedures. Participant IDs uniquely identified each participant and remained consistent throughout the study.

### 2.5. Study Procedures and Endpoint Hierarchy

Once eligibility was confirmed by the study physicians, sociodemographic characteristics, medical history, baseline WURSS‐21, VAS score, and NRS score for symptom severity were collected from participants. Subsequently, blood samples were collected for routine baseline laboratory investigations, after which participants were enrolled and randomized by blinded researchers. Participants reported to the clinical site on Day 1, Day 2, Day 3, and Day 5 for clinical evaluation. Laboratory testing included assessments of hematological and biochemical parameters, such as CBC, serum creatinine, SGPT, SGOT, lipid profile, RBS, uric acid, and urinalysis. Inflammatory and immune biomarkers were analyzed at baseline and Day 3, including CRP levels in blood, as well as IL‐8 and IgA levels in nasal wash samples, to assess immune response and inflammation.

To ensure statistical and clinical clarity, a clear hierarchy of endpoints was established. The primary clinical efficacy endpoint was defined as the change in uncomplicated URTI symptom severity and functional impact, quantified via the validated WURSS‐21. Secondary exploratory endpoints included patient‐reported pain scales via VAS and NRS, objective nasal mucosal weight tracking (g), and localized inflammatory biomarkers.

### 2.6. Statistical Analysis

Continuous variables were characterized using descriptive statistics, including sample size (*n*), mean, standard deviation (SD), median, minimum, and maximum values. Statistical analysis was performed using SPSS software (Version 29.0.1.0 or similar), maintaining a significance level of 5%.

Within‐group analyses for WURSS, VAS, NRS, and nasal mucosal weight were conducted using repeated‐measures analysis of variance (ANOVA), with Bonferroni‐adjusted pairwise comparisons for post hoc analysis. Where the assumption of sphericity was violated, as evaluated by Mauchly′s test, Greenhouse–Geisser′s corrections were applied. Effect sizes (partial eta squared) with 95% confidence intervals were reported for repeated‐measures ANOVA to quantify the magnitude of within‐group changes.

For between‐group comparisons, independent‐samples *t*‐tests were utilized to evaluate differences at the postdose (6‐h) assessment endpoint; note that baseline values were inherently integrated into this initial postdose evaluation timeframe, precluding a separate baseline imbalance analysis. Equality of variances was assessed using Levene′s test. The total cumulative disease burden over the study duration was determined by calculating the AUC for WURSS‐21 parameters using the standard trapezoidal rule, with subsequent between‐group AUC differences evaluated using independent‐samples *t*‐tests. Within‐group changes in biomarker parameters were assessed using paired‐samples *t*‐tests, whereas between‐group differences were evaluated using independent‐samples t‐tests. For independent‐samples *t*‐tests, effect sizes (Cohen′s) with 95% confidence intervals were reported to quantify the magnitude and precision of between‐group differences.

## 3. Results

### 3.1. Participants

**Figure 1:**
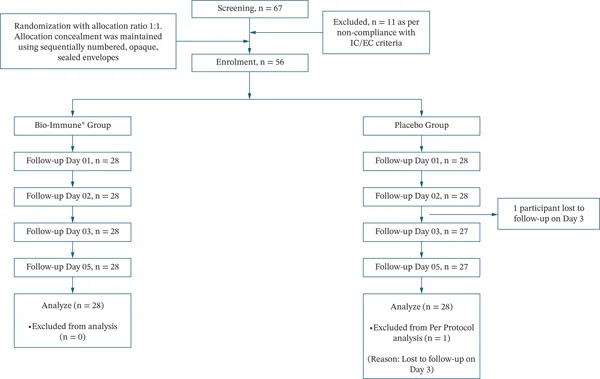
A CONSORT flowchart depicting the progression of participants throughout the intervention period of the clinical trial. *n*: no. of participants.

A total of 67 participants were initially screened to ensure at least 25 participants in each group. Of these, 56 (18 females and 38 males) were randomized, and 55 completed the study. In one group, 28 participants received Bio‐Immune 100 mg twice daily, whereas in another group, 28 participants received placebo twice daily for 5 consecutive days. Figure 1 represents a CONSORT flowchart depicting the progression of participants throughout the intervention period of the clinical trial. New participants were not recruited to replace dropouts. Due to the high completion rate (98.2%), a per‐protocol analysis was performed, evaluating only the 55 participants who successfully completed the 5‐day intervention period. The single participant who withdrew from the trial was excluded from the final efficacy and statistical analyses, eliminating the requirement for missing data imputation. The mean age of participants in the Bio‐Immune group was 40.96 (SD ±6.91), whereas the mean age of the placebo group was 43.32 (SD ±7.17). Approximately 68% of participants were men, whereas 32% of participants were female, enrolled during the clinical study.

### 3.2. Primary Outcome Measures

#### 3.2.1. Changes in WURSS‐21 Score

The Bio‐Immune group showed a significant reduction in symptom severity score compared with the placebo group at all assessed time points, as shown in Table 2. Starting from comparable baseline scores, the Bio‐Immune group experienced statistically significant improvements by 6 h after administration (*p* < 0.001, mean change of −3.57 ± 3.14), achieving near‐complete symptom improvement by Day 5 (mean score of 0.21 ± 0.69). By contrast, the placebo group showed no significant improvement at the 6‐h mark (*p* = 0.495) and, despite gradual longitudinal improvements through Day 5, retained a noticeably higher residual symptom burden (mean score of 4.48 ± 2.42). Direct between‐group comparisons confirm that Bio‐Immune maintained significant superiority over the placebo at every postbaseline time point (*p* < 0.05 across all intervals), with the most substantial supplement advantage peaking on Day 3 (effect estimate = −1.52; 95% CI: [−2.12, −0.91]).

**Table 2:** A comparative analysis of WURSS‐21 score, VAS score, NRS score, and nasal mucosal weight measures between groups.

Time point		Bio ‐Immune®					Placebo					*Between Group comparison*			
		Mean	SD (±)	*p-value*	*Effect size*	*95% CI*	Mean	SD (±)	*p-value*	*Effect size*	*95% CI*	*p-value*	*Effect size*		*95% CI of effect size*
													*Standardiser*	*Estimate*	
**Symptoms Severity Score (WURSS-21)**															
Baseline (Day 1)	OV	12.93	4.11	—	0.88	—	11.07	4.28	—	0.59	—	—	—	—	—
Post 6 h (Day 1)	OV	9.36	2.53	<0.001		[1.76,5.39]	10.63	4.68	0.495		[−0.22,1.11]	<0.05	2.38	−1.32	[−1.90, −0.73]
	CFB	−3.57	3.14				−0.44	1.12							
Day 2	OV	4.61	1.97	<0.001		[6.19,10.45]	9.52	4.73	0.013		[0.23,2.88]	<0.05	3.06	−2.21	[−2.88, −1.53]
	CFB	−8.32	3.68				−1.56	2.24							
Day 3	OV	2.61	1.34	<0.001		[7.94,12.71]	7.07	3.15	<0.001		[1.53,6.47]	<0.05	4.15	−1.52	[−2.12, −0.91]
	CFB	−10.32	4.13				−4.00	4.18							
Day 5	OV	0.21	0.69	<0.001		[10.5,14.93]	4.48	2.42	<0.001		[4.59,8.59]	<0.05	3.62	−1.69	[−2.30, −1.07]
	CFB	−12.71	3.83				−6.59	3.39							
**Functional Impairment Score (WURSS-21)**															
Baseline (Day 1)	OV	7.18	3.33	—	0.79	—	5.7	2.35	—	0.70	—	—	—	—	—
Post 6 h (Day 1)	OV	4.75	2.49	<0.001		[1.04,3.82]	5.67	2.39	1		[−0.08,0.15]	<0.05	1.73	−1.39	[−1.97, −0.79]
	CFB	−2.43	2.41				−0.04	0.19							
Day 2	OV	1.50	1.53	<0.001		[4.02,7.34]	4.52	2.03	0.0301		[0.07,2.3]	<0.05	2.43	−1.85	[−2.47, −1.21]
	CFB	−5.68	2.87				−1.19	1.88							
Day 3	OV	0.18	0.48	<0.001		[5.18,8.82]	2.96	2.21	<0.001		[1.08,4.4]	<0.05	2.99	−1.43	[−2.01, −0.83]
	CFB	−7.00	3.15				−2.74	2.81							
Day 5	OV	0.00	0.00	<0.001		[5.25,9.1]	0.41	0.64	<0.001		[3.96,6.63]	<0.05	2.86	−0.66	[−1.20, −0.11]
	CFB	−7.18	3.33				−5.3	2.27							
**Global Score (WURSS-21)**															
Baseline (Day 1)	OV	20.11	6.29	—	0.88	—	16.78	5.65	—	0.71	—	—	—	—	—
Post 6 h (Day 1)	OV	14.11	4.32	<0.001		[3.18,8.82]	16.3	6.01	0.3463		[−0.18,1.14]	<0.05	3.57	−1.55	[−2.15, −0.94]
	CFB	−6.00	4.88				−0.48	1.12							
Day 2	OV	6.11	3.13	<0.001		[10.65,17.35]	14.04	5.74	0.0079		[0.53, 4.95]	<0.05	4.90	−2.30	[−2.98, −1.61]
	CFB	−14.00	5.79				−2.74	3.75							
Day 3	OV	2.79	1.60	<0.001		[13.8,20.85]	10.04	5.03	<0.001		[3.02,10.46]	<0.05	6.20	−1.71	[−2.32, −1.08]
	CFB	−17.32	6.10				−6.74	6.30							
Day 5	OV	0.21	0.69	<0.001		[16.35,23.44]	4.89	2.79	<0.001		[9.23,14.55]	<0.05	5.40	−1.48	[−2.08, −0.88]
	CFB	−19.89	6.14				−11.89	4.50							
**Total VAS Score**															
Baseline (Day 1)	OV	152.57	30.78	—	0.92	—	137.41	34.38	—	0.77	—	—	—	—	—
Post 6 h (Day 1)	OV	119.93	34.70	<0.001		[15.84,49.44]	126.89	34.86	0.001		[3.42,17.62]	<0.05	22.40	−0.99	[−1.54, −0.42]
	CFB	−32.64	29.08				−10.52	12.03							
Day 2	OV	60.61	34.55	<0.001		[73.45,110.48]	110.33	38.74	<0.001		[12.58,41.57]	<0.05	28.62	−2.27	[−2.94, −1.58]
	CFB	−91.96	32.05				−27.07	24.56							
Day 3	OV	28.07	20.10	<0.001		[109.29,139.71]	83.37	37.52	<0.001		[33.75,74.32]	<0.05	30.54	−2.31	[−2.99, −1.61]
	CFB	−124.50	26.33				−54.04	34.37							
Day 5	OV	1.50	4.54	<0.001		[134.95,167.19]	54.04	35.27	<0.001		[63.45,103.29]	<0.05	30.91	−2.19	[−2.86, −1.51]
	CFB	−151.07	27.91				−83.37	33.75							
**Total NRS Score**															
Baseline (Day 1)	OV	16.64	7.09	—	0.83	—	14.15	5.52	—	0.62	—	—	—	—	—
Post 6 h (Day 1)	OV	12.32	5.12	<0.001		[2.52,6.13]	13.41	4.85	0.0511		[0,1.48]	<0.05	2.40	−1.49	[−2.09, −0.89]
	CFB	−4.32	3.13				−0.74	1.26							
Day 2	OV	6.21	3.32	<0.001		[7.61,13.25]	11.37	4.88	0.0017		[0.83,4.72]	<0.05	4.18	−1.83	[−2.46, −1.19]
	CFB	−10.43	4.89				−2.78	3.3							
Day 3	OV	3.04	1.82	<0.001		[9.81,17.4]	8.56	3.95	<0.001		[2.25,8.93]	<0.05	6.14	−1.31	[−1.88, −0.72]
	CFB	−13.61	6.57				−5.59	5.66							
Day 5	OV	0.21	0.69	<0.001		[12.53,20.33]	5.41	2.79	<0.001		[5.63,11.85]	<0.05	6.07	−1.27	[−1.84, −0.68]
	CFB	−16.43	6.75				−8.74	5.27							
**Nasal Mucosal Weight Measures**															
Baseline (Day 1)	OV	2.11	0.92	—	0.6	—	2.14	0.62	—	0.51	—	—	—	—	—
Day 2	OV	1.65	0.82	0.0884		[−0.04,0.96]	1.88	0.51	0.046		[0,0.52]	0.7823	0.82	−0.27	[−0.45, 0.60]
	CFB	−0.46	0.93				−0.26	0.47							
Day 3	OV	1.36	0.74	<0.001		[0.32,1.19]	1.53	0.38	<0.001		[0.35,0.87]	0.2861	0.75	−0.22	[−0.24, 0.82]
	CFB	−0.75	0.81				−0.61	0.48							
Day 5	OV	0.41	0.21	<0.001		[1.18,2.24]	1.11	0.43	<0.001		[0.55,1.51]	0.0298	0.92	−0.72	[−1.14, −0.06]
	CFB	−1.71	0.98				−1.03	0.88							

*Note:* Within‐group variations were evaluated via repeated‐measures ANOVA with the Greenhouse–Geisser and Bonferroni corrections as appropriate. Between‐group variations were evaluated via independent‐samples *t*‐tests. Statistical significance is defined at *p* < 0.05.

Abbreviations: CFB, change from baseline; CI, confidence interval; NRS, Numerical Rating Scale; OV, observed value; SD, standard deviation; VAS, Visual Analogue Scale; WURSS‐21, Wisconsin Upper Respiratory Symptom Survey‐21.

Similarly, the Bio‐Immune group demonstrated a significant reduction in functional impairment score (WURSS‐21) and showed improvement in quality of life compared with the placebo group at all assessed time points, as shown in Table 2. From baseline values (7.18 ± 3.33 for the active group vs. 5.70 ± 2.35 for the placebo group), the active group demonstrated immediate, highly significant functional improvement by 6 h after administration (*p* < 0.001, mean change from baseline of −2.43 ± 2.41), ultimately achieving complete resolution of functional impairment by Day 5 (mean score of 0.00 ± 0.00). By contrast, the placebo group exhibited virtually no improvement at the 6‐h mark (*p* = 1.000, mean change of −0.04 ± 0.19) and, while showing significant progress from Day 2 onward (*p* = 0.0301 on Day 2; *p* < 0.001 by Days 3 and 5), still retained residual impairment by Day 5 (mean score of 0.41 ± 0.64). Direct between‐group comparisons confirm that the active intervention maintained absolute statistical superiority (*p* < 0.05) at every postbaseline observation point, with the treatment benefit peaking on Day 3 (effect estimate = −1.43; 95% CI: [−2.01, −0.83]).

With regard to the global score (WURSS‐21), the Bio‐Immune group showed a significant reduction in global score compared with the placebo group, as shown in Table 2. From baseline values (20.11 ± 6.29 for the active group vs. 16.78 ± 5.65 for the placebo group), the active group demonstrated immediate, highly significant functional improvement by 6 h after administration (*p* < 0.001, mean change from baseline of −6.00 ± 4.88), with global symptom burdens tracking down to near‐complete improvement by Day 5 (mean score of 0.21 ± 0.69). By contrast, the placebo group exhibited virtually no improvement at the 6‐h mark (*p* = 0.3463, mean change of −0.48 ± 1.12) and, while showing significant progress from Day 2 onward (*p* = 0.0079 on Day 2; *p* < 0.001 by Days 3 and 5), sustained a notably higher residual symptom burden by the end of the study period (mean score of 4.89 ± 2.79). Direct between‐group comparisons confirm that the active intervention maintained absolute statistical superiority (*p* < 0.05) at every postbaseline observation point, with the treatment benefit peaking on Day 3 (effect estimate = −1.71; 95% CI: [−2.32, −1.08]).

### 3.3. AUC Analysis for WURSS‐21

The AUC for WURSS‐21 scores is a method used to summarize the overall burden of illness over time, integrating both the intensity and duration of symptoms. Total cumulative scores (refer to Tables 3 and 4) across the study period were substantially lower in the Bio‐Immune arm across all metrics, showing a significant overall reduction in symptom severity (total AUC: 514.39 ± 159.36 vs. 964.67 ± 389.72), functional impairment (total AUC: 191.46 ± 117.65 vs. 418.67 ± 178.02), and global WURSS‐21 burden (total AUC: 705.86 ± 242.85 vs. 1383.33 ± 501.84), with all overall between‐group comparisons reaching high statistical significance (*p* < 0.001). Although interval subanalyses showed no distinct treatment variations at the early Day 1 (6‐h) mark (*p* > 0.05), a powerful and sustained therapeutic divergence emerged by Day 2 and persisted through Day 5, with the active treatment continually demonstrating absolute statistical superiority over the placebo (*p* = 0.003 at Day 2 and *p* < 0.001 at Days 3 and 5 across all parameters).

**Table 3:** A comparative analysis of WURSS‐21 AUC scores between groups on Day 5.

	Bio‐Immune (*n* = 28)		Placebo (*n* = 27)		*p* *v*alue between groups
	Mean	SD (±)	Mean	SD (±)	
Symptom severity score AUC	514.39	159.36	964.67	389.72	< 0.001
Functional impairment score AUC	191.46	117.65	418.67	178.02	< 0.001
Global WURSS‐21 score AUC	705.86	242.85	1383.33	501.84	< 0.001

Abbreviations: AUC, area under the curve; SD, standard deviation; WURSS‐21: Wisconsin Upper Respiratory Symptom Survey‐21.

**Table 4:** Between‐group analysis for WURSS‐21 AUC score—confidence interval.

Parameter	Time points	Mean difference between groups	Std. error of the difference between groups	95% CI	Two‐sided *p* value
Symptom severity score AUC	Day 1 (6 h)	1.75	6.18	[−10.7, 14.19]	0.779
	Day 2 (48 h)	−129.86	40.51	[−212.03, −47.69]	0.003
	Day 3 (72 h)	−112.54	17.42	[−147.89, −77.19]	< 0.001
	Day 5 (120 h)	−209.62	24.83	[−260.22, −159.02]	< 0.001
	Total AUC	−450.27	80.82	[−614.49, −286.06]	< 0.001

Functional impairment score AUC	Day 1 (6 h)	1.67	4.09	[−6.53, 9.88]	0.684
	Day 2 (48 h)	−82.64	21.65	[−126.09, −39.19]	< 0.001
	Day 3 (72 h)	−69.63	9.82	[−89.52, −49.75]	< 0.001
	Day 5 (120 h)	−76.60	11.46	[−100.07, −53.13]	< 0.001
	Total AUC	−227.20	40.84	[−309.47, −144.93]	< 0.001

Global score AUC	Day 1 (6 h)	3.42	8.65	[−13.94, 20.78]	0.694
	Day 2 (48 h)	−212.50	52.49	[−318.39, −106.61]	< 0.001
	Day 3 (72 h)	−182.17	24.21	[−231.25, −133.09]	< 0.001
	Day 5 (120 h)	−286.22	33.44	[−354.54, −217.9]	< 0.001
	Total AUC	−677.48	106.93	[−894.09, −460.86]	< 0.001

*Note:* The AUC curve was calculated using the trapezoidal formula, and the *p* value was calculated using an independent *t*‐test.

Abbreviations: AUC, area under the curve; CI, confidence interval; WURSS‐21: Wisconsin Upper Respiratory Symptom Survey‐21.

### 3.4. Secondary Outcome Measures

#### 3.4.1. VAS Score

The Bio‐Immune group demonstrated a significant reduction in VAS score compared with the placebo group at all assessed time points, as represented in Table 2. From comparable baseline values (152.57 ± 30.78 for the active group vs. 137.41 ± 34.38 for the placebo group), the active group demonstrated immediate, highly significant improvements by 6 h after administration (*p* < 0.001, mean change from baseline of −32.64 ± 29.08), dropping continuously to a near‐complete resolution by Day 5 (mean score of 1.50 ± 4.54). Although the placebo group also showed statistically significant longitudinal improvements across all postbaseline time points (*p* = 0.001 at 6 h; *p* < 0.001 thereafter), their recovery was remarkably slower, leaving a substantial residual burden by Day 5 (mean score of 54.04 ± 35.27). Direct between‐group comparisons demonstrate that the active group maintained clear statistical superiority (*p* < 0.05) at every single evaluation point, with the maximum treatment benefit expanding progressively up to Day 5 (effect estimate = −2.19; 95% CI: [−2.86, −1.51]).

#### 3.4.2. NRS Score

The Bio‐Immune group also demonstrated a significant reduction in NRS score compared with the placebo group at all assessed time points, as represented in Table 2. From baseline scores of 16.64 ± 7.09 for the active arm and 14.15 ± 5.52 for the placebo arm, the active group experienced a rapid and highly significant alleviation of symptoms by 6 h (*p* < 0.001, mean change of −4.32 ± 3.13), resolving almost entirely by Day 5 (mean score of 0.21 ± 0.69). In contrast, the placebo group failed to reach statistical significance at the post‐6‐h evaluation (*p* = 0.0511, mean change of −0.74 ± 1.26) and, despite showing meaningful longitudinal progress from Day 2 onward (*p* = 0.0017), remained significantly more symptomatic by Day 5 (mean score of 5.41 ± 2.79). Direct between‐group comparisons validate that the active intervention was significantly superior (*p* < 0.05) at all postbaseline intervals, with the largest comparative treatment advantage achieved on Day 3 (effect estimate = −1.31; 95% CI: [−1.88, −0.72]).

#### 3.4.3. Nasal Mucosal Weight and Biomarkers

Table 2 summarizes the between‐group differences in nasal mucosal weight (g), whereas Table 5 details nasal wash IL‐8 (pg/mL) and serum hsCRP (mg/L). There was a trend toward a difference in nasal mucosal weight between the Bio‐Immune and placebo groups, and it achieved statistical significance on Day 5. From nearly identical baseline values (2.11 ± 0.92 for the active group vs. 2.14 ± 0.62 for the placebo group), the active group did not initially exhibit a significant reduction on Day 2 (*p* = 0.0884) but went on to show highly significant within‐group reductions on Day 3 (*p* < 0.001, mean change of −0.75 ± 0.81) and Day 5 (*p* < 0.001, mean change of −1.71 ± 0.98). In contrast, the placebo group demonstrated a minor significant change on Day 2 (*p* = 0.046) and followed a slower downward trajectory through Day 5 (*p* < 0.001, mean score of 1.11 ± 0.43). Although direct between‐group comparisons did not show a statistically significant difference on Day 2 (*p* = 0.7823) or Day 3 (*p* = 0.2861), the active group established a clear, statistically significant therapeutic superiority by Day 5 (*p* = 0.0298), culminating in a markedly lower mucosal weight measure compared with the placebo cohort (effect estimate = −0.72; 95% CI: [−1.14, −0.06]).

**Table 5:** A comparative analysis of CRP and IL‐8 measures between groups.

Parameters |Time Point		Bio‐Immune®						Placebo						*Between Group comparison*			
		Mean	SD (±)	*p-value*	*Effect size*		*95% CI*	Mean	SD (±)	*p-value*	*Effect size*		*95% CI*	*p-value*	*Effect size*		*95% CI of effect size*
					*Standardizer*	*Point Estimate*					*Standardizer*	*Point Estimate*			*Standardizer*	*Point Estimate*	
CRP (mg/L)	**Baseline Day 1**	5.71	1.98	0.1430	0.79	−0.29	[−0.53, 0.08]	7.25	7.69	0.6464	4.02	−0.09	[−1.95, 1.23]	0.8660	2.87	0.05	[−0.48, 0.58]
	**Day 3**	5.48	1.76					6.89	4.86								
	**CFB**	−0.23	0.79					−0.36	4.02								
Nasal wash IL8 (pg/ml)	**Baseline Day 1**	15.71	14.77	0.2629	15.20	−0.22	[−9.18, 2.61]	14.96	9.85	0.3015	7.85	0.20	[−4.70, 1.51]	0.6048	12.17	−0.14	[−0.67, 0.39]
	**Day 3**	12.43	5.72					13.37	8.20								
	**CFB**	−3.29	15.20					−1.59	7.85								

*Note:* Within‐group variations were evaluated using paired *t*‐tests. Between‐group variations were evaluated via independent‐samples *t*‐tests. Statistical significance is defined at *p* < 0.05.

Abbreviations: CFB, change from baseline; CI, confidence interval; CRP, C‐reactive protein; IL‐8, Interleukin‐8; SD, standard deviation.

Inflammatory biomarkers between the Bio‐Immune and placebo groups for either serum hsCRP or nasal wash IL‐8 at any postbaseline time point were also evaluated (Table 5). The small numerical reductions from baseline (Day 1 to Day 3) observed in the parameters lacked adequate statistical support (*p* > 0.05), indicating that a demonstrable localized or systemic anti‐inflammatory effect cannot be confirmed within this short 3‐day observation window. There were no changes observed in the IgA biomarker value in both study groups.

No adverse events were reported during the study. Laboratory parameters, including CBC, serum creatinine, SGPT, SGOT, lipid profile, RBS, uric acid, and urine physical examination (pH and specific gravity), remained within normal ranges in both groups after intervention.

## 4. Discussion

The study is aimed at evaluating the preliminary exploratory efficacy of Bio‐Immune at a lower dose of 100 mg, twice daily (containing andrographolide ≥ 17% *w*/*w*), in managing symptoms and functional impairment associated with uncomplicated URTI. A significant reduction in WURSS‐21, VAS, and NRS scores was observed by 6 h after supplementation with Bio‐Immune on Day 1, compared with the placebo group, indicating that Bio‐Immune helps improve symptom severity and functional impairment in a shorter duration. Statistically significant changes across all three scores were observed by 6 h after supplementation in the Bio‐Immune group, suggesting an encouraging preliminary trend toward early symptom relief. This early divergence was confirmed by between‐group statistical evaluation at the post‐6‐h assessment point, suggesting a measurable acceleration in early symptom relief shortly after the initial dose, establishing a foundation for the accelerated recovery observed over the subsequent Day 3 and almost complete recovery by Day 5. The placebo group experienced significant gradual symptom reduction, with fewer symptoms remaining on Day 5. Although the Bio‐Immune cohort achieved maximum improvement in both symptom severity and functional impairment scores by the third day, the placebo cohort merely demonstrated a slow, day‐by‐day natural progression. This significant divergence indicates that while both groups would eventually resolve due to the self‐limiting nature of the condition, the intervention dramatically accelerates the timeline of clinical resolution and minimizes the cumulative daily disease burden.

A significant reduction in nasal mucosal secretion was observed on Day 5 in the Bio‐Immune group compared with the placebo group. Inflammatory biomarkers have been evaluated in the Bio‐Immune group, showing a minor numerical reduction in CRP and IL‐8 from baseline Day 1 to Day 3 compared with the placebo group, but these changes were not statistically significant (*p* > 0.05). Consequently, a definitive localized or systemic anti‐inflammatory mechanism of action cannot be confirmed or supported by the given data within this short 3‐day observation window and small sample size. There were no changes found in the value of the IgA biomarker in both groups. The observed reduction in nasal mucus weight over time, along with nonsignificant minor reductions in CRP and IL‐8 alongside stable IgA levels, reflects a favorable reduction in localized symptomatic secretion without causing disruptive systemic inflammation or compromising baseline mucosal immunity.

Overall study results indicate that oral supplementation with Bio‐Immune significantly reduces symptoms associated with uncomplicated URTI, dramatically accelerates the timeline of clinical resolution, and minimizes the cumulative daily disease burden. No adverse events were reported during the study. The findings of this study suggest Bio‐Immune as a potential ingredient in the nutraceuticals or health supplements category.

A limitation of this clinical study is that it was conducted initially at a pilot scale to confirm the effective dose of Bio‐Immune (100 mg, twice daily) for managing symptoms associated with uncomplicated URTI. Additionally, this small‐scale, short‐duration, single‐center study restricted the study population to participants aged ≥ 30 years. Further studies using Bio‐Immune at a 100 mg twice‐daily dose in large‐scale, fully powered, multicenter confirmatory trials encompassing a broader adult age spectrum (18+ years) and extended posttreatment tracking are required to definitively validate these clinical observations.

## 5. Conclusion

Bio‐Immune is a standardized extract containing a bioactive compound, andrographolide (≥ 17% *w*/*w*), extracted from *A. paniculata*, with improved bioavailability, solubility, and half‐life [9]. In this pilot exploratory study, twice‐daily oral supplementation with a lower dose of Bio‐Immune (100 mg) was well tolerated and demonstrated encouraging preliminary trends in mitigating URTI symptom severity and functional impairment. A statistically detectable early divergence in symptom scores was noted at the 6‐h postsupplementation assessment window, followed by an accelerated reduction in overall symptom burden over the 5‐day timeline compared with the placebo group. No adverse events were reported in either cohort. Given the exploratory nature, small sample size, and single‐center study, these findings will form the basis for initiating a larger scale, robust clinical trial across multiple sites.

**Nomenclature**
RCT: randomized controlled trial. A clinical trial where participants are randomly assigned to different groups to compare the effects of interventions, reducing selection bias.
Double‐blinded: Both the participants and investigators are unaware of group assignments to prevent bias in treatment administration or outcome assessment.
Placebo‐controlled: A study where the intervention group receives the active substance and the control group receives an inert placebo to allow for comparison.
Single‐center study: A study conducted at one clinical research site only.
URTI: upper respiratory tract infection. An infection of the nose, sinuses, pharynx, or larynx, commonly presenting as the common cold, sore throat, or similar ailments.
Andrographolide: A diterpenoid lactone isolated from Andrographis paniculata, considered the primary bioactive molecule responsible for therapeutic effects.
2‐HPBCD: 2‐hydroxypropyl‐*β*‐cyclodextrin. A chemically modified cyclodextrin that enhances the water solubility, stability, and bioavailability of active compounds.
Diterpenoid lactone: A class of organic compounds with a specific ring structure, often contributing to biological activities such as anti‐inflammatory effects.
CRP: C‐reactive protein. A protein produced by the liver, used as a marker for systemic inflammation or infection.
IL‐8: Interleukin‐8. A cytokine involved in inflammation, promoting the recruitment of immune cells to infection sites.
IgA: Immunoglobulin A. An antibody important for mucosal immunity, providing defense at the respiratory and other mucosal surfaces.
WURSS‐21: Wisconsin Upper Respiratory Symptom Survey‐21. A validated questionnaire measuring symptom severity and functional impairment during URTI.
VAS: Visual Analogue Scale. A 100 mm continuous scale where participants rate the intensity of symptoms from “*no symptoms*” to “*worst imaginable symptoms*.”
NRS: Numeric Rating Scale. An 11‐point scale ranging from 0 (*no symptoms*) to 10 (*worst possible symptoms*), used for subjective symptom assessment.
AUC: area under the curve. A quantitative measure that integrates symptom intensity over time, reflecting overall illness burden.
Nasal mucus weight: The total mass of nasal secretions collected over time, used as an objective indicator of congestion severity.
Bioavailability: The fraction of an administered dose that reaches systemic circulation and is available to exert a therapeutic effect.
Half‐life: The time it takes for the concentration of a substance in the body to decrease by 50%.
NOAEL: no observed adverse effect level. The maximum dose at which no harmful effects are observed in toxicity studies.
LD^50^
: lethal dose for 50% of the population
CBC: complete blood count
SGPT: serum glutamic‐pyruvic transaminase
SGOT: serum glutamic‐oxaloacetic transaminase
RBS: random blood sugar
CRO: contract research organization
CTRI: Clinical Trial Registry of India
NIH: National Institutes of Health
CI: confidence interval

## Author Contributions

**Shashi Chandrama Singh:** conceptualization, data curation, formal analysis, investigation, methodology, project administration, writing – original draft, review and editing. **Muskan Choudhary:** conceptualization, funding acquisition, methodology, project administration, resources. **Khushi Choudhary:** conceptualization, funding acquisition, investigation, methodology, project administration, resources, software, supervision. **Harsh Pal Singh:** conceptualization, funding acquisition, methodology, resources, software, supervision, validation, writing – review and editing.

## Funding

This study was funded by AMBE PHYTOEXTRACTS PVT LTD.

## Ethics Statement

This study was reviewed and approved by the Clinic Research Ethics Committee–ACEAS Independent Ethics Committee registered at CDSCO and OHRP US DHHS, with CDSCO registration number ECR/281/Indt/GJ/2017/RR‐21 and OHRP US DHHS registration number IRB00011046. At CTRI (Clinical Trial Registry of India), this clinical trial has been registered (on December 4, 2024). It has also been registered at ClinicalTrials.gov (registry of clinical trials, run by the US National Library of Medicine [NLM] at the National Institutes of Health [NIH]). All participants signed a written informed consent form before participating in the clinical trial study.

## Conflicts of Interest

The authors declare the following competing financial interest: All authors are employees of Ambe Phytoextracts Pvt. Ltd., the company that manufactured the product evaluated in this study and funded this research.

## Data Availability

The data that support the findings of this study are available on request from the corresponding author.
